# Effects of a video-viewing intervention with positive word stimulation on the depressive symptoms of older patients with cardiac disease and subthreshold depression: a pilot randomized controlled trial protocol

**DOI:** 10.1186/s13030-024-00312-w

**Published:** 2024-07-16

**Authors:** Masataka Sakimoto, Takumi Igusa, Takuya Kobayashi, Hiroyuki Uchida, Aya Fukazawa, Chihaya Machida, Hirokuni Fujii, Keisuke Sekine, Minori Kurosaki, Kenji Tsuchiya, Senichiro Kikuchi, Kazuki Hirao

**Affiliations:** 1 Graduate School of Health Sciences, Gunma University, 3-39-22 Showa, Maebashi, Gunma 371-8514 Japan; 2 Department of Rehabilitation, Fujioka General Hospital, Fujioka, Japan; 3 Department of Rehabilitation, Medical Corporation Taiseikai, Uchida Hospital, Numata, Japan; 4 Department of Rehabilitation, Japan Community Healthcare Organization, Gunma Chuo Hospital, Maebashi, Japan; 5 Department of Rehabilitation, Kurashiki Heisei Hospital, Kurashiki, Japan; 6 Department of Rehabilitation, Faculty of Health Sciences, Nagano University of Health and Medicine, Nagano, Japan

**Keywords:** Subthreshold depression, Cardiac disease, Older adults, Mental health

## Abstract

**Background:**

Intervention for older patients with cardiac disease and subthreshold depression (StD) may be an effective strategy to prevent the development of major depressive disorder. The subliminal priming with supraliminal reward stimulation (SPSRS) website developed by us is an advanced intervention that can improve depressive symptoms in individuals with StD by presenting positive word stimuli in videos. However, its efficacy for treating depressive symptoms in older patients with cardiac disease and StD has not been investigated. Here, we present a pilot randomized controlled trial protocol to investigate the preliminary efficacy of an intervention for older patients with cardiac disease with StD.

**Methods:**

The study was designed as a single-center, open-label, pilot, randomized, parallel-group trial. The participants will include 30 older patients with cardiac disease and StD who are hospitalized in acute wards. The Experimental group received the SPSRS intervention (video viewing with positive word stimuli; n = 15) and the Control group will receive the YouTube intervention (video viewing without positive word stimuli; n = 15). In both groups, the intervention will be administered for 10 min per day, five times per week for 1 week. The primary outcome will be the change in the scores on the Japanese version of the Beck Depression Inventory-II at 1 week after the baseline assessment. The secondary outcomes will be the changes in the Specific Activity Scale, New York Heart Association functional classification, as well as grip strength at 1 week after the baseline assessment.

**Discussion:**

This pilot randomized controlled trial will be the first to evaluate the SPSRS intervention for depressive symptoms in older patients with cardiac disease and StD who are admitted to acute wards. The results will provide tentative indications regarding the impact of the intervention on depressive symptoms among older patients with cardiac disease and StD who are admitted to acute wards, and will contribute to the planning of a full-scale study.

**Trial registration:**

UMIN, UMIN000052155. Registered September 8, 2023, https://center6.umin.ac.jp/cgi-open-bin/ctr/ctr_view.cgi?recptno=R000059526. This study was registered with the University Hospital Medical Information Network (UMIN) (UMIN000052155) in Japan.

## Background

Both cardiac disease (e.g., coronary artery disease, heart failure, heart valve disease, and arrhythmia) and major depressive disorder (MDD) are prevalent worldwide and are projected to be major contributors to the global burden of disease through 2030, according to the World Health Organization [1]. Moreover, both conditions are important public health issues. MDD in patients with cardiac disease is a significant burden and a particularly important problem [2]. The prevalence of MDD among patients with cardiac disease is 15%–20% [3, 4], which is approximately twice that of the nonhospitalized general population [5]. Of particular importance, it has been suggested that cardiac disease and depression mutually increase each other’s risk [6], causing behavioral changes, such as decreased medication adherence [7], poor exercise capacity [8], and physical inactivity [9]. Therefore, this has been suggested to cause increased blood pressure, variability in heart rate, and arrhythmias [10, 11]. It is widely reported that MDD in patients with cardiac disease leads to increased cardiovascular events, more severe cardiac disease, decreased quality of life, mortality, and increased medical costs [12–18].

The aging of the population compounds these problems. As of 2019, the world population over 65 years of age was 703 million, and this figure is projected to increase to 1.5 billion by 2050, i.e., one out of every six people in the world [19], especially in developed countries, where the population is aging rapidly [20]. The American Heart Association predicts a significant increase in the prevalence of cardiac disease because of advanced age by 2030 [21]. Although the estimated prevalence of MDD among older adults is quite low (1%–4%) [22], it has been suggested that the prevalence of MDD in older adults admitted for medical treatment is 10%–12%, with an additional 23% of these individuals experiencing significant depressive symptoms [23]. This may accelerate the increase in the prevalence of MDD among older patients with cardiac disease, resulting in increased mortality, reduced quality of life, and increased health care costs.

Several intervention strategies have been proposed to address the various problems caused by MDD in older patients with cardiac disease. However, it has been suggested that MDD is less likely to be detected in this group of patients [24–26]. In addition, it has been suggested that approximately one-third of patients who are generally recognized to have MDD and receive treatment for it do not respond to current approaches, and more than half of those who develop MDD for the first time will have one or more relapses [27]. In particular, psychological and pharmacological interventions for cardiac disease populations have been reported to have significantly lower effect sizes compared with those observed for other chronic diseases, such as diabetes [28–30]. Furthermore, large trials, such as the landmark Enhancing Recovery in Coronary Heart Disease Patients study, have questioned the methodology and acceptability of MDD treatment in the population of patients with heart disease [31], as MDD treatment in these patients did not result in a significant reduction in major adverse cardiac events [32]. Given the high disease burden of MDD in these older patients with cardiac disease, the lack of awareness, and the ineffectiveness of current therapies, it is critical to implement early detection of, and preventive treatment for, at-risk patients. Preventive intervention for subthreshold depression (StD), which is a precursor symptom of MDD, may be an effective and cost-efficient approach [33–36].

Although StD does not meet the diagnostic criteria for MDD, it is characterized by clinically significant depressive symptoms [37]. It has been suggested that the prevalence of StD among patients with cardiac disease ranges from 17.0% to 27% [38–40]. Moreover, the number of people with StD is increasing in the older population [41, 42]. Even though StD is a precursor of MDD, it is known to cause decreased quality of life [37, 43], increased mortality [44, 45], poor health status [46], and increased health care costs [47]. Of particular importance is the fact that StD is a risk factor for the development of MDD [48, 49]. In fact, previous studies have suggested that 42% of patients with cardiac disease and StD will transition to MDD [39]. For these reasons, intervention strategies for StD should be established, and their effectiveness in preventing MDD development should be investigatedin older patients with cardiac disease and StD. To address the problems associated with StD, several preventive intervention strategies have been proposed to improve depressive symptoms specifically in patients with cardiac disease and StDs [50–52].

The use of antidepressants and other medications is common for treating MDD [53]. In addition, it has been suggested that, for patients with StD, the use of pharmacotherapy can have a positive impact by promoting an improvement in the overall depressive symptoms [53]. Conversely, a systematic review that investigated the effects of pharmacotherapy on StD did not find a significant reduction in depressive symptoms compared with the placebo [54]. In addition, it has been suggested that drug therapy can cause a variety of problems, including sexual dysfunction, hypotension, dizziness, insomnia, and appetite changes [55]. In addition, it has been proposed that the use of pharmacotherapy also increases the risk of myocardial infarction and other adverse events in patients with cardiac disease [56, 57]. Furthermore, patients with cardiac disease may have lower rates of medication adherence [7]. These problems regarding drug therapy may limit its use in older patients with cardiac disease and StD. Exercise therapy for MDD may also be an effective intervention to improve depressive symptoms [58, 59]. In addition, exercise therapy for patients with cardiac disease and MDD may reduce depressive symptoms [60, 61], and may decrease depressive symptoms in patients with StD [62]. However, it may be difficult from a medical safety standpoint to implement aggressive exercise therapy and other measures among older patients immediately after the onset of cardiac disease. Because of the problems associated with these existing interventions, interventions that are specifically designed to reduce depressive symptoms in older patients with cardiac disease and StD are needed. Therefore, the video replay website called subliminal priming with supraliminal reward stimulation (SPSRS) developed by us may be an effective intervention strategy to reduce depressive symptoms in older patients with cardiac disease and StD [63].

SPSRS is a video playback website that displays positive word stimuli in videos to improve depressive symptoms in patients with StD [63, 64]. The SPSRS tool features a website that allows patients to access it, search for videos by entering keywords, and watch the videos. In addition, SPSRS is free of charge and uses the YouTube Application Programming Interface, which allows patients to select from a wide variety of videos of interest and work toward reducing depressive symptoms. Previous studies have suggested that the use of SPSRS among individuals with StD compared with no intervention not only improves their depressive symptoms [64], but may also improve depressed mood immediately after the 10-min intervention [65]. In addition to the existing studies showing the effectiveness of SPSRS interventions in reducing depressive symptoms in patients with StD, one of the major factors that renders SPSRS applicable to older patients with cardiac disease is that it allows them to work toward reducing depressive symptoms without moving from their bed. Previous studies have suggested that video viewing can be performed with relatively low physical effort (1.0 to 1.5 Mets) when performed in the supine or end-sitting position [18, 66]. Therefore, SPSRS may be a safe approach to reduce depressive symptoms in older patients with cardiac disease who have difficulty performing high exercise loads. Given the limitations of these existing interventions (potentially for adverse events and difficulty of implementation) and the potential of SPSRS (potentially reducing depressive symptoms in people with StD and the relatively low loadings of 1.0–1.5 Mets that can be introduced), a randomized controlled trial (RCT) investigating the efficacy of SPSRS in older patients with cardiac disease and StD. However, no interventional studies have been conducted to examine the effects of SPSRS among older patients with cardiac disease and StD.

The Medical Research Council methodological framework was adopted to evaluate the effectiveness of the SPSRS intervention in older patients with cardiac disease and StD [67]. This methodology includes development, feasibility, piloting, evaluation, and implementation phases [67]. The feasibility and piloting phases are presented in the present study. Pilot studies are the best approach to assess the feasibility of larger, more expensive trials. Moreover, conducting pilot studies before larger trials helps to inform on the optimal design and scale and increases the likelihood of success [68]. Therefore, the purpose of this study was to present a protocol aimed at investigating the preliminary efficacy of the SPSRS intervention for depressive symptoms among older patients with cardiac disease and StD.

## Materials and methods

### Trial design and study setting

The study was designed as a single-center, open-label, pilot, randomized, parallel-group trial. The participants in this study will be older patients with cardiac disease and StD who are admitted to acute wards. We will compare the effects of a video-viewing intervention using the SPSRS (with positive word stimulation) and a video-viewing intervention using YouTube (without positive word stimulation). The Fujioka General Hospital is a public hospital that specializes in the treatment and rehabilitation of disuse syndrome, musculoskeletal disorders, cerebrovascular disorders, respiratory disorders, cardiovascular disorders, cancer, and pediatric disorders. The acute wards of the Fujioka General Hospital provide individual therapy performed by physical therapists (PTs), occupational therapists (OTs), and speech therapists (STs) to promote activities of daily living and independence to leave home among these patients. The acute wards at the Fujioka General Hospital provide individual therapy delivered by PTs, OTs, and STs for a maximum of 6–9 units per day (1 unit = 20 min), 7 days per week, 365 days per year. All patients admitted to these acute wards will be assigned a specific PT, OT, or ST. An alternate PT, OT, or ST may provide rehabilitation services when the assigned PT, OT, or ST is absent or unable to intervene. To prevent biases associated with the study design, this protocol followed the Standard Protocol Items: Recommendations for Interventional Trials (SPIRIT) 2013 statement [69].

### Eligibility criteria

Those who meet the following eligibility requirements will be selected as participants in the study.

#### Inclusion criteria

1. Men and women.
2. Patients admitted to the acute wards of the Fujioka General Hospital.
3. Individuals aged 65 years or older.
4. Patients with cardiac disease (coronary artery disease, heart failure, heart valve disease, and arrhythmia).
5. Patients with a total score of 10 or more on the Japanese version of the Beck Depression Inventory-II [70].
6. Persons with sufficient cognitive ability to understand the content and purpose of the research.
7. Persons who provided written consent before participation in this study.
8. Patients with New York Heart Association functional classification (NYHA classification) degrees I–III.

#### Exclusion criteria

1. Individuals who have been diagnosed with a mental disorder at least once in their lifetime, regardless of the type of mental disorder.
2. Patients currently receiving professional treatment for mental health issues.
3. Individuals with visual or hearing impairments that interfere with daily living.
4. Patients assessed by the Mini-international Neuropsychiatric Interview (M.I.N.I.) as having had a major depressive episode in the last 2 weeks [71].
5. Patients with serious life-threatening complications (severe organ failure, respiratory disorders, cerebrovascular disorders, and musculoskeletal disorders).

The assessment of auditory, visual, and cognitive function, as well as impairment for the study is at the discretion of the therapist [72]. These parameters will be determined based on the patient’s response to verbal instructions during the administration of the Japanese version of the Mini-Mental State Examination (MMSE-J) and other assessments, and on the patient’s ability to recognize the objects used [72].

### Interventions

#### Experimental group

**Fig. 1:**
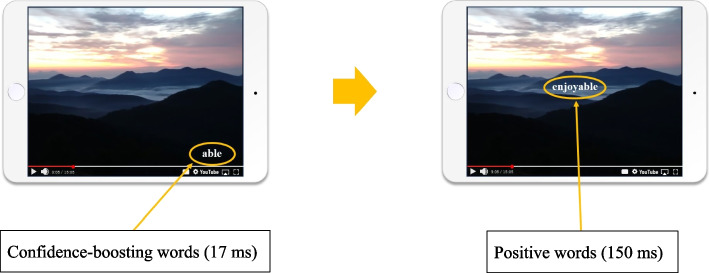
SPSRS intervention

**Fig. 2:**
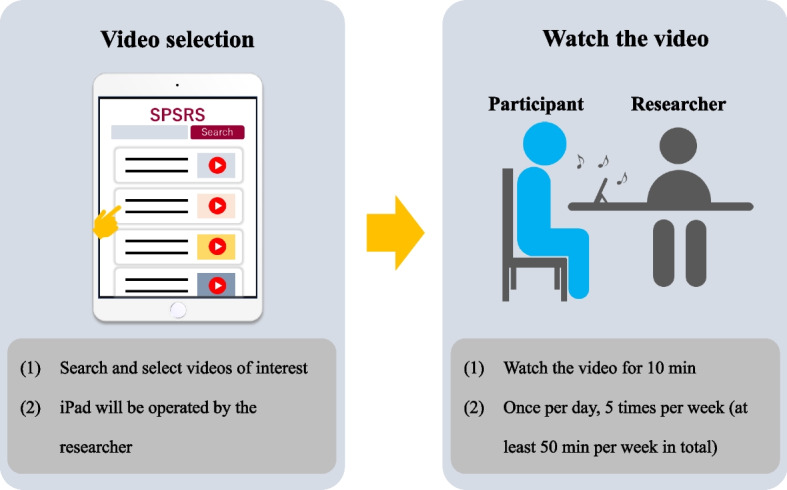
Protocols for the SPSRS intervention

The Experimental group will receive a video-viewing intervention using SPSRS. The SPSRS tool is designed to present confidence-boosting words in the video for 17 ms, followed by 150 ms of positive words (Fig. 1). The confidence-boosting words are “can,” “let us try,” “good luck,” “able,” and “do not worry,” which appear randomly in the four corners of the screen [73]. The positive words are “nice,” “great,” “fantastic,” “satisfactory,” and “enjoyable,” and appear randomly in the center of the screen [74]. These words are presented repeatedly every 5 s. An iPad will be used as the medium for video viewing, which will be conducted with the researcher in attendance. SPSRS is used according to the operating manual. After accessing the SPSRS website using a researcher-controlled iPad, the participants will search for videos of interest and watch a 10-min video in the presence of the researcher (Fig. 2). If there is no video that the participant wants to watch, the researcher will suggest a video and watch it with the participant’s approval. The participants will watch the 10-min videos once per day, five times per week for a total intervention time of at least 50 min. The video-viewing time in this study was determined based on a previous study that examined the effects of the SPSRS intervention on patients with StD [64].

#### Control group

The participants in the Control group will receive a total of at least 50 min of intervention per week, with 10 min of video viewing once per day, five times per week, for a total of at least 50 min per week, after accessing the YouTube website using a researcher-controlled iPad to search for videos of interest to them. If there is no video that the participant wants to watch, the researcher will suggest a video and watch it with the participant’s approval, as described for the Experimental group. The Control group videos will not show confidence-boosting or positive words; the Control group will use the same iPad as did the Experimental group.

### Criteria for discontinuing or modifying the allocated interventions

The research team will discontinue the intervention if any of the following conditions are met by the research subject:

1. If the research subject wishes to discontinue participation in the research.
2. If the research team determines that it is difficult to continue the study because of deterioration of the mental or physical health of the patient.
3. If the entire clinical study is terminated.
4. If it is discovered after enrollment that a participant does not meet eligibility.
5. If the study team determines that the intervention should be discontinued for any reason.

The date and reason for discontinuation will be fully documented in the case report form. Case reports will provide information on the feasibility and acceptability of the intervention, to improve the standard practice of SPSRS interventions for future full-scale trials. The participants will not be considered to have dropped out of the study upon discontinuation of the intervention; rather, they will be asked to undergo primary and secondary outcome assessments. If a participant refuses to undergo the evaluation or withdraws consent, the participant will be considered as having dropped out of the study. Participants will be excluded from the intention-to-treat (ITT) analysis only if they are found to be unable to meet the eligibility criteria.

### Relevant concomitant care and interventions permitted or prohibited during the trial

This study will not prohibit other forms of care, from an ethical standpoint.

### Outcomes

The primary outcome will be the change in the scores on the Japanese version of the BDI-II at 1 week after the baseline assessment [75]. In turn, the secondary outcomes will be the changes in the minimum amount of exercise at which heart failure symptoms appear on the Specific Activity Scale (SAS) [76], New York Heart Association functional classification (NYHA classification) [77], as well as grip strength at 1 week after the baseline assessment.

### Recruitment timeline

**Fig. 3:**
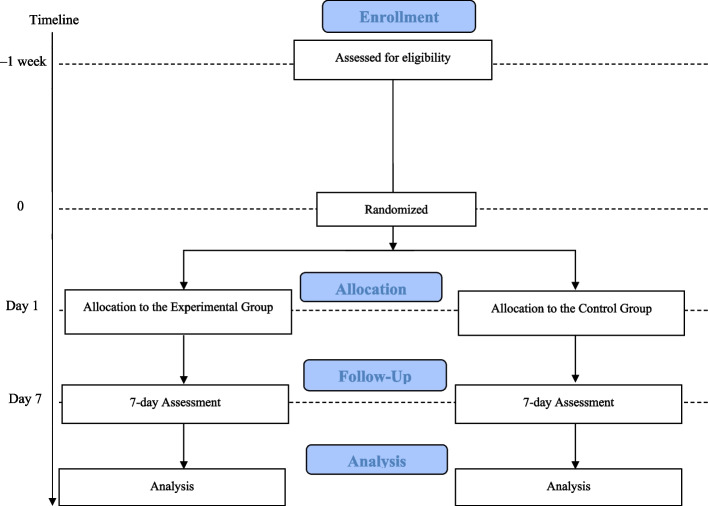
CONSORT flowchart of the study design

The study will be conducted at the Fujioka General Hospital in Gunma, Japan, from October 2023 to March 2027. The researcher will distribute a brochure with a brief description of the study to potential participants, to encourage their participation. Potential participants will be assessed for eligibility through interviews and questionnaires, and enrolled in the study after baseline assessments (BDI-II and SAS, NYHA classification, and grip strength assessment). The baseline and eligibility assessments will be performed by the researcher within 1 week of admission to the acute wards. The participants will be randomly assigned to the Experimental or Control group after the eligibility and baseline assessments. Both groups will then receive a 10-min video-viewing intervention once per day, five times per week for 1 week. The participants will undergo remeasurements for second BDI-II and SAS, NYHA classification, and grip strength at 1 week after the intervention. A flowchart of the study design is shown in Fig. 3 [78]. The assessment schedule is provided in Table 1 [79].

**Table 1:** Assessment schedule

			**STUDY PERIOD**		
	**Enrolment**	**Allocation**	**Baseline**	**Intervention**	
TIMEPOINT**	***–1 week***	**0**	***0***	***1 day***	***7 days***
**Enrolment**					
**Eligibility screen**	X				
**Informed consent**	X				
**Allocation**		X			
**Interventions:**					
***Experimental group***					
***Control group***					
**Assessments:**					
***Demographics***	X				
***BDI-II***			X		X
***SAS***			X		X
***NYHA classification***			X		X
**Grip strength**			X		X

* BDI-II* Beck Depression Inventory-II

*SAS* Specific Activity Scale

*NYHA classification* New York Heart Association classification

### Sample size

There are no data on SPSRS interventions for patients with cardiac disease and StD admitted to acute wards, which hampers the estimation of the sample size based on actual data. Although a formal sample size calculation is not required in a pilot study [68], a sample size of 15–20 persons should be maintained in a pilot study for conducting a subsequent full-scale study [80]. Therefore, the goal of this study was to recruit 15 participants per group, for a total of 30 participants.

### Allocation

The participants will be randomly assigned to the Experimental or Control group in a 1:1 ratio after the eligibility criteria check and baseline assessment. Using a computer (Excel software), a randomized list will be generated using the permuted block method. To ensure allocation concealment, the block sizes will not be disclosed until after trial completion. The generation of the randomized list will be performed by a third party not involved in the study. Throughout this process, the participants, evaluators, and intervention implementers will be denied access to information that might reveal the assignments. The randomized list will be provided to the Central Registration Center located at Gunma University, for random assignment.

### Blinding

It will be difficult to blind the study participants and intervention providers because the Experimental and Control group interventions are distinctly different. In addition, the small number of research staff who will participate in this study renders it difficult to maintain rater blinding. Therefore, this study is planned as an open-label trial.

### Data collection

Standard operating procedures and evaluation guides for data collection and storage will be provided to the Fujioka General Hospital, and a 2-h training session will be offered. Meetings will also be held regularly to discuss issues related to the implementation of the trial. The assessments of the primary and secondary outcomes will be conducted according to the schedule provided in Table 1. Age, gender, primary disease at admission, MMSE-J score [81], left ventricle ejection fraction, and blood laboratory data (albumin, hemoglobin, creatinine, and brain natriuretic peptide levels) will be collected as the baseline assessment. In addition, the types of videos viewed by both groups were collected.

#### Mini-mental State Examination (MMSE-J)

The MMSE-J consists of 10 items that assess orientation, registration, attention or calculation, recall, naming, repetition, comprehension, reading, writing, and construction, respectively [81]. Lower MMSE-J scores indicate a more severe cognitive impairment, with total scores ranging from 0 to 30 points. MMSE-J is a reliable and validated assessment that is widely used clinically [82].

#### Mini-international Neuropsychiatric Interview (M.I.N.I.)

The M.I.N.I. is a structured diagnostic interview based on the DSM-IV and ICD-10. The Japanese version of M.I.N.I. is designed to encompass 16 psychiatric diagnostic modules. Respondents can answer “Yes” or “No” exclusively. The reliability and validity of the M.I.N.I. have been reported in previous studies [71, 83].

#### Beck Depression Inventory-II (BDI-II)

The BDI-II is a 21-item self-administered questionnaire that measures the severity of depression symptoms. Items are rated using a 4-point Likert scale ranging from 0 to 3 points [84]. The total score ranges from 0 to 63 points, with higher scores indicating more depressive symptoms. The reliability and validity of the BDI-II have been reported in previous studies [75, 84, 85]. This study used BDI-II to identify the presence of StD in older patients with cardiac disease and to measure changes in depressive symptoms during a 1-week intervention. This is because BDI-II, in addition to being widely used to identify StD, is also widely used to assess depressive symptoms in clinical populations, including patients with cardiac disease [65, 70, 86–89].

#### Specific Activity Scale (SAS)

The SAS lists specific activities of daily living for which exercise intensity (metabolic equivalents: Mets) is mostly known in advance, and participants are asked to answer “Yes,” “No,” or “unknown” in order of the activity with the lowest physical activity index. If the patient answers “Yes,” the question gradually shifts to higher activity, and the point at which the patient answers “No” is the point of the minimum amount of exercise (Met) at which heart failure symptoms appear is calculated [76]. The validity and reliability of the SAS have been reported in previous studies [90].

#### New York Heart Association functional classification

The NYHA cardiac function classification was developed by the New York Heart Association to classify the severity of cardiac disease according to the degree of subjective symptoms observed after physical activity and is widely used as a severity classification in the context of heart failure [77]. This classification includes grade I (presence of cardiac disease but no limitation of physical activity), grade II (mild to moderate limitation of physical activity on exertion, asymptomatic at rest), grade III (severe limitation of physical activity on exertion, asymptomatic at rest), and grade IV (cardiac disease that limits any physical activity and presence of heart failure symptoms and anginal pain even at rest) (77).

#### Grip strength

Grip strength will be measured using a digital grip strength dynamometer (T.K.K. 5401 GRIP-D; Takei, Japan). The measurement will be performed in the sitting position, with the shoulder joint placed at 0° and the elbow joint placed in extension, twice on each side. The maximum value of the results obtained will be used in the analysis. Grip strength is correlated with the overall muscle strength [91]. The reliability of the measuring instruments has been reported [92, 93].

#### Plan to promote participant retention and completion of the follow-up

The study planners will inform the participants about the details of the study schedule and the importance of the study to prevent participant dropout and facilitate follow-up [94]. Furthermore, all interventions and outcome assessments will be performed free of charge. In addition, outcome assessments and information collection will be minimized, to reduce the burden on the hospitalized patients. Participants who meet the criteria for refusal to continue the study or for discontinuation of the intervention will be invited to participate in a postintervention evaluation. These suggestions will be highlighted and explained to the participants during informed consent.

### Data management, confidentiality, and access

To ensure data accuracy, the participants’ data will first be transcribed onto paper. Two independent researchers will then enter the data into an Excel spreadsheet. These data entries will be performed on a computer that is not connected to the Internet. All information entered will be stored on a password-protected USB stick. The next step will be the checking of the collected data by researchers other than the two researchers who entered the data for accuracy, missing data, and data consistency [94]. The paper data created previously will be stored in a lockable storage facility at Gunma University, and the electronic data will be stored in a separate lockable cabinet. Backups of electronic data will be created on hard drives that are not connected to the Internet. The participants’ data will be anonymized and managed using random codes. These data will be stored for 10 years after the publication of the article and will be properly erased after the storage period. Only the principal investigator and data manager will have access to the collected data. The principal investigator and data manager will investigate data issues and prepare the dataset for statistical analysis. After publication, only the principal investigator and selected groups of individuals will have access to the dataset. No computer at Gunma University that is connected to the Internet will be used for statistical analysis.

### Statistical methods

The ITT principles will be followed during the analysis of all assigned participant data. The primary and secondary outcomes will be compared between the two groups using linear mixed models with a restricted maximum-likelihood estimation method for repeated measurement analysis. Group, time, and their interaction will be used as fixed-effect variables, whereas the participants will be used as random-effect variables. All significance levels will be set at *P* < 0.05 using two-tailed tests. No subgroup analysis is planned at this time. All analyses will be performed using the latest version of SPSS. The effect size (Hedge’s g) between groups will also be calculated [95, 96].

### Data monitoring and auditing

At the present time, no formal data oversight committee has been established and no data audit is planned. Furthermore, no interim analysis of the impact of the intervention is planned.

### Ethics and dissemination (approval, protocol amendments, and consent)

This study was approved by the Ethical Review Board for Medical Research Involving Human Subjects of Gunma University (approval number: HS2023-050). The study was also approved by the Ethical Review Committee of Fujioka General Hospital (approval number: 338). Before participation in the study, the subjects will be briefed by the principal investigator or a research collaborator on the study via written research briefing materials. Any changes to the research plan must be approved by the Ethical Review Board for Medical Research Involving Human Subjects of Gunma University and the Ethical Review Committee of Fujioka General Hospital. Before enrolling the participants in the study, the researcher will explain the benefits and risks of participation, ensure that the participants are aware of the benefits and risks of the intervention, and obtain consent to participate. Participants who agree to participate in the study must provide written informed consent.

### Ancillary and posttrial care

The participants may contact the researcher at any time during the study period. After the 1-week intervention, the researchers will continue to track the progress of the participants and document the changes in their physical and mental health. There is no guarantee that unforeseen problems will not arise during or after the study. In the event of serious complications or health problems, the necessary treatment will be provided within standard insurance coverage. The cost of treatment will be borne by the participant, as is the case with regular treatment.

### Dissemination policy

The results of this research, regardless of their validity, will be submitted to peer-reviewed journals. In addition, the research results and their scientific significance will be reported and disseminated at academic conferences.

## Discussion

This study is a pilot RCT aimed at investigating the effect of the SPSRS intervention on depressive symptoms in older patients with cardiac disease and StD who are admitted to acute wards. StD is a risk factor for increased mortality, incidence, rehospitalization rates, and severity of illness among older patients with cardiac disease. However, it has been suggested that the existing treatments, such as pharmacotherapy, for older patients with cardiac disease and StD do not adequately reduce depressive symptoms [32, 97–99]. Therefore, there is a need to identify alternative approaches for treating depressive symptoms in older patients with cardiac disease. The SPSRS website may be a new intervention strategy that can improve depressive symptoms in older patients with cardiac disease and StD. The SPSRS is a video-viewing website in which positive language stimuli are presented, and can be accessed through any Internet-enabled device. Therefore, the SPSRS intervention is less burdensome for the patients and interveners; has a low cost; is simple, novel, and guaranteed to be of similar quality for all participants; and can provide treatment to various individuals. Therefore, we designed a pilot RCT to investigate the preliminary efficacy of the SPSRS intervention among older patients with cardiac disease and StD. This pilot RCT was designed to enhance the scientific validity of full-scale RCTs. The results of this study will provide additional insights into the preliminary effectiveness and feasibility of the SPSRS intervention, and will allow us to identify the modifications and improvements that are necessary for a successful larger RCT.

This study has several strengths. First, this is the first pilot RCT to investigate the preliminary efficacy of the SPSRS intervention for improving depressive symptoms in older patients with cardiac disease and StD who are admitted to acute wards. This pilot RCT may lead to the development of a state-of-the-art treatment approach to improve depressive symptoms in older patients with cardiac disease presenting with StD, and can expand our knowledge of the benefits and limitations of intervention strategies for the management of depressive symptoms in this specific population. Second, the study has a high external validity, because it includes patients suffering from a variety of cardiac diseases.

This study also had several limitations. First, this study will be conducted on an open-label basis because of its nature. This may lead to a bias among the treatment providers, study participants, and evaluators. When interpreting the study results, it will be necessary to consider the effects of other factors, such as participant expectations and the relationship between the treatment provider and the patient, on depressive symptoms. Second, because this is the first study to examine the effect of the SPSRS intervention on older patients with cardiac disease and StD who are admitted to acute wards, no formal sample size calculations will be performed. This limitation may reduce the statistical power of the primary and secondary outcomes. Third, because this pilot RCT will be conducted at a single site exclusively, the sample is less representative, and not all findings obtained will apply to older patients with cardiac disease and StD who are admitted to acute wards [100]. Fourth, this study may underestimate the effects of SPSRS intervention on depressive symptoms in older patients with cardiac disease and StD. The intervention duration in this study was 1 week because the number of days of hospitalization should be considered in the acute care of patients with cardiac disease in Japan and the feasibility of data collection and the duration of possible rehabilitation intervention. The BDI-II is a self-report measure of depressive symptoms that assesses symptoms over the past 2 weeks; however, previous studies indicate that a 1-week intervention period can produce significant differences in the BDI-II [87–89, 101, 102]. For these reasons, we employed BDI-II in this study. However, considering that the BDI-II is a self-report measure of depressive symptoms that assesses symptoms over the past 2 weeks, we cannot dismiss the possibility that the intervention period of this study (one week) will result in little changes in depressive symptoms as measured by BDI-II. Finally, because the specific video to be viewed during the SPSRS is determined based on participant self-selection from a full range of videos, the differences in the participants’ video choices may have an impact on depressive symptoms.

## Conclusions

This pilot RCT is the first study to investigate the impact of an SPSRS intervention on depressive symptoms in older patients with cardiac disease and StD. This pilot RCT, which was designed according to an appropriately standardized procedure, will allow the evaluation of the potential of a larger trial to detect the effects of the SPSRS intervention on depressive symptoms in older patients with cardiac disease and StD. If the results of the intervention are positive, the SPSRS intervention may also become a new strategy to improve depressive symptoms in older patients with cardiac disease and StD.

## Data Availability

Not applicable.
